# Real-World Characterization and Treatment Patterns of Patients with Desmoid Tumors at an Academic Center in the United States

**DOI:** 10.1158/2767-9764.CRC-25-0581

**Published:** 2026-04-09

**Authors:** Chia Jie Tan, Connor Willis, Timothy Bell, Brad Tumminello, Shengfan Zhou, Dipam Doshi, Carl V. Asche, Joseph Biskupiak, Diana Brixner, Anna Chalmers, David D. Stenehjem

**Affiliations:** 1 Department of Pharmacotherapy, College of Pharmacy, University of Utah, Salt Lake City, Utah.; 2 SpringWorks Therapeutics, Inc., Stamford, Connecticut.; 3 Huntsman Cancer Institute, Salt Lake City, Utah.; 4 Department of Pharmacy Practice and Pharmaceutical Sciences, College of Pharmacy, University of Minnesota, Duluth, Minnesota.

## Abstract

Real-world studies on desmoid tumors (DT) are limited due to its low incidence. Therefore, we aimed to characterize the patient journey in DTs from diagnosis to treatment at an academic center in the United States. A retrospective cohort study was conducted at the University of Utah Health network. Eligible patients were diagnosed with DTs between January 1, 2011, and July 31, 2023, were ≥18 years old at diagnosis, and had ≥2 DT-related encounters. Patient and tumor characteristics, reports of symptoms, and treatment patterns were abstracted from electronic health records. Healthcare resource utilization was quantified using administrative claims. Patients who potentially had a misdiagnosis ≤2 years before DT diagnosis were identified based on billing codes of conditions commonly diagnosed instead of DTs. Among 148 eligible patients, 59.5% had documentation of ≥1 DT-related symptom during the follow-up period. Pain was the most common reported symptom (57.4%). 79.7% of patients received ≥1 line of active treatment; some received up to 10 lines of therapy. Healthcare resource utilization was higher during periods of symptomatic disease and among patients with potential misdiagnosis (29.1%) in the year before DT diagnosis. Although surgery was the most common treatment, its use decreased across the study period with a corresponding expansion of systemic treatment modalities used as first-line treatment. In conclusion, increased healthcare resource utilization associated with misdiagnosis highlights the need for timely and accurate DT diagnosis. The varied treatment approaches and multiple lines of therapy suggest the need for optimal therapeutic agents. Surgery rates have decreased, aligning with evolving treatment guidelines.

**Significance:**

DT remains an understudied disease area despite its high morbidity. Due to limited data on treatment patterns and outcomes, particularly in the United States, gaps in the management of DTs are not well elucidated. Therefore, we aimed to describe the patient journey in DTs within our health system.

## Introduction

Desmoid tumors (DT) are locally aggressive tumors of soft tissues, with a tendency to infiltrate surrounding structures (1). Although they do not metastasize, DTs are associated with local recurrence rates ranging from 24% to 77% after surgical resection, regardless of margin status (2). Because of the difficulty in achieving effective treatment in many cases, the disease course of DTs is typically protracted. Constituting less than 0.03% of all neoplasms with 3 to 5 new cases per million individuals per year, DTs are considered a rare disease (3). However, the risk of DTs is highly elevated in selected patient populations, such as individuals with familial adenomatous polyposis (FAP; ref. 4). Patients with DTs have reported pain that can be debilitating and nonresponsive to analgesics (5). Additionally, DTs are associated with functional limitations, such as restricted limb movement, that negatively affect the ability to perform daily activities, leading to impaired quality of life (5). Among patients with FAP, DTs are typically larger and more aggressive, potentially leading to life-threatening complications, such as bowel obstruction and bowel perforation (6).

Surgical resection has historically been the primary intervention for DTs. However, this approach has been associated with high recurrence rates (2, 7). Additionally, extensive surgery may be required to achieve optimal control of DTs, which can result in significant patient burden, including disfigurement and functional impairment, particularly in limb-associated DTs (2, 5, 7). Over the past decade, treatment recommendations for DTs have evolved. As similar event-free survival has been observed between patients who received surgery and watchful waiting as initial management approaches for DTs, active surveillance is now recommended for asymptomatic or minimally symptomatic, nonmorbid or nonprogressive DTs (8–10). If treatment is warranted, systemic treatment options, such as γ secretase inhibitors, tyrosine kinase inhibitors (TKI), and cytotoxic chemotherapy should be considered first-line treatment (8, 9). In general, surgery is no longer recommended as a first-line treatment option in most clinical scenarios, and the Desmoid Tumor Working Group in 2015, 2017, 2020 and 2024 recommended systemic treatment modalities as alternatives to surgery as first-line options for active intervention, with the exception of abdominal wall tumors (8, 9, 11–13).

Nirogacestat, a γ secretase inhibitor approved in the United States on November 27, 2023, is currently the only Food and Drug Administration (FDA)-approved therapy for adult patients with progressing DTs who require systemic treatment based on findings from the pivotal trial DeFi (14). Prior to the approval of nirogacestat, TKIs were the primary options for systemic treatment of DTs. Sorafenib has demonstrated superior progression-free survival over placebo in a randomized controlled trial, whereas imatinib and pazopanib have shown progression-free survival rates of 66% and 86% at 1 year, respectively, in nonrandomized or noncomparative studies (15–19). Both nirogacestat and sorafenib are category 1 treatment options in the NCCN Clinical Practice Guidelines in Oncology (NCCN guidelines; ref. 13).

Despite the high morbidity associated with DTs, it remains an understudied disease area due to its low incidence rate. There are limited data on real-world treatment patterns and outcomes of patients with DTs, particularly in the United States (2). For this reason and due to the transforming treatment landscape, we aim to describe the patient journey in DTs by illustrating symptom burden, treatment patterns, and healthcare resource utilization, including a subgroup analysis of patients with misdiagnoses prior to DT diagnosis.

## Materials and Methods

A retrospective cohort study was conducted via chart review of electronic medical records within the University of Utah Health network, an academic center serving the Mountain West region, including Utah, Idaho, Montana, Nevada, and Wyoming. Part of the network includes the Huntsman Cancer Institute (HCI), a high-volume referral center for patients with sarcoma. The study was conducted in accordance with the Helsinki Declaration. Due to its retrospective nature and chart review approach, this study was exempted from review by the University of Utah Institutional Review Board (IRB), and written informed consent was waived (IRB_00168804).

### Study population

The study population of interest was patients diagnosed with DTs. To be eligible for the study, patients had to be (i) diagnosed with DTs between January 1, 2011, and July 31, 2023, (ii) at least 18 years old at diagnosis of DTs, and (iii) documented to have at least two encounters (i.e., interaction in which healthcare services were provided, including outpatient clinic visits and inpatient hospitalization episodes) related to the patient’s DT management to exclude those who were not undergoing care for DTs within the University of Utah Health network and would have incomplete outcomes data.

Patients who had potentially been diagnosed with DTs were first identified using International Classification of Disease (ICD) Clinical Modification (CM) billing codes 238.1 (ninth version) or D48.1 (10th version), which represent neoplasms of uncertain behavior of connective and other soft tissue. However, as these codes were not specific for DTs, we also retrieved cancer registry records with ICD-Oncology tumor site codes indicating benign soft tissue tumors or fibromatosis and clinical notes that contained the keywords “desmoid” or “fibromatosis” between January 1, 2011, and July 31, 2023. A DT diagnosis was confirmed by review of pathology reports (i.e., histology of biopsy specimen was consistent with DTs) and clinical notes.

### Study variables

Structured data, such as demographic characteristics and billing data, were directly retrieved from the University of Utah Health data warehouse, whereas unstructured data, including reports of symptoms, treatment patterns, and documentation of disease progression, were manually abstracted from clinical notes, pathology reports, and imaging reports. Patients were followed from the diagnosis of DTs to the date of last encounter within the University of Utah Health network. The date of DT diagnosis was inferred from explicit documentation in the clinical notes by a physician, confirmation of histology consistent with DTs in a pathology report by a pathologist or the earliest episode of care coded with 238.1 or D48.1, by order of priority. Study variables that were collected and quantified included the following:

#### Patient characteristics at diagnosis of DTs

Baseline demographic and clinical characteristics of patients at diagnosis of DTs were extracted. Demographic characteristics included age, sex, race and ethnicity, insurance status, and state of residence. Clinical characteristics included the size of the primary DT, disease focality, tumor location, catenin β-1 (*CTNNB1*) and adenomatous polyposis coli (*APC*) mutation status, diagnosis of FAP, and history of other cancers. The primary DT referred to the first tumor detected at diagnosis, or if more than one tumor were detected, the tumor that was largest in size. Patient characteristics were presented as medians/interquartile ranges (IQR) for continuous variables and number of patients and percentages for categorical variables.

#### Disease activity

Disease progression was inferred from terms documented by healthcare providers in the clinical notes during DT-related encounters (Supplementary Table S1). Symptomatic disease was ascertained based on documentation of symptoms, including tumor-related pain, paresthesia, fatigue, swelling, muscle weakness, loss of function (such as difficulty in moving limbs or walking), nausea, and fullness. Patients were considered to have active disease if disease progression or symptomatic disease was reported or active treatment was ongoing. The proportion of patients with at least one episode of disease progression, symptomatic disease, or active disease was quantified.

#### Treatment patterns

All DT-related treatment was recorded from diagnosis until the end of the study period (July 31, 2023, prior to the FDA approval of nirogacestat), including the type of treatment, line of therapy, dates of initiation, and dates of discontinuation. Treatments that were recorded included surgery, radiotherapy, local ablative procedures, cytotoxic chemotherapy, TKIs, and tamoxifen. A new line of therapy was defined as a distinct change in treatment regimen due to disease progression, adverse side effects, or patient/clinician preference. Dates of initiation and discontinuation were abstracted from clinical notes and verified by medication administration or pharmacy records if necessary. The proportion of patients who received each type of treatment was quantified by line of therapy. Additionally, the median time to recurrence (i.e., interval between surgery date and date of first documented progression) was estimated for patients who received surgery using Kaplan–Meier methods, censoring for death, the last encounter in the University of Utah Health network, or the end of the study period, whichever occurred earliest.

#### Healthcare resource utilization

Healthcare resource utilization included outpatient visits, emergency room visits, and hospitalization episodes. Only healthcare resource utilization linked to ICD-CM codes related to DTs (238.1 and D48.1) were included in our analysis. The proportion of patients with at least one episode of healthcare resource utilization was measured. The median rates of outpatient and emergency room visits were estimated on a per-patient per-year basis by averaging the number of visits for each patient over the duration of study follow-up. The length of hospitalization was calculated based on admission and discharge dates.

#### Potential misdiagnosis prior to DT diagnosis

Patients who were potentially misdiagnosed prior to an official diagnosis of DTs were identified using ICD-CM codes associated with other conditions or disorders that are commonly misdiagnosed in place of DTs (Supplementary Table S2). The characteristics of patients who were potentially misdiagnosed and healthcare resource utilization within 1 and 2 years prior to official DT diagnosis were reported.

### Statistical analysis

Changes in overall first-line treatment patterns and use of individual treatment modalities over time were assessed using Jonckheere–Terpstra tests for trend and Cochran–Armitage tests for trend with Bonferroni correction to adjust for multiple testing, respectively. Characteristics between (i) patients who received first-line systemic and local treatment modalities and (ii) those with misdiagnosis and those without were compared using Wilcoxon rank-sum tests for continuous variables and *χ*^2^ tests/Fisher exact tests for categorical variables. The proportion of patients with visits and median healthcare resource utilization rates between those with misdiagnosis and those without were compared using *χ*^2^ tests and Wilcoxon rank-sum tests, respectively. Statistical analysis was performed using STATA version 18 (RRID: SCR_012763).

## Results

### Patient characteristics

A total of 148 patients were eligible for this study with a median follow-up time (from the time of DT diagnosis to the last documented DT-related encounter) of 26.9 months (IQR: 12.5–61, range: 1.1–144.5). Patient characteristics are described in Table 1, and the patient flow diagram is shown in Supplementary Fig. S1. The median age at diagnosis was 36 years (IQR: 27–46, range: 18–78), with the majority being female (*n* = 101, 68.2%). At baseline, the median size of the primary tumor was 60 mm (IQR: 40–92, 11–180). DTs were more likely to be unifocal (*n* = 128, 86.5%), and the most common locations of the primary tumor were the chest wall (*n* = 42, 28.4%), abdominal wall (*n* = 41, 27.7%), and intraabdominal region (*n* = 29, 19.6%).

**Table 1.** Baseline demographics and clinical characteristics of patients at DT diagnosis.

Characteristic	*N* = 148
Age at diagnosis, mean (SD)	38.1 (14.1)
Age at diagnosis, median (IQR, range)	36 (27–46, 18–78)
Sex, *n* (%)	​
Male	47 (31.8%)
Female	101 (68.2%)
Race and ethnicity, *n* (%)^a^	​
Asian	3 (2%)
Black/African American	3 (2%)
Hispanic/Latino	11 (7.4%)
Native American/Alaska Native	1 (0.7%)
White	131 (88.5%)
Others	0 (0%)
Chose not to disclose/undocumented	5 (3.4%)
Insurance status, *n* (%)	​
Commercial	118 (79.7%)
Medicare	10 (6.8%)
Medicaid	10 (6.8%)
VA/Tricare	5 (3.4%)
Uninsured	5 (3.4%)
Geographic region, *n* (%)	​
Utah	90 (60.8%)
Non-Utah	58 (39.2%)
Primary DT size in mm, mean (SD)	67.8 (36.9)
Primary DT size in mm, median (IQR, range)	60 (40–92, 11–180) (Unreported = 9)
Primary DT location, *n* (%)^b^	​
Abdominal wall	41 (27.7%)
Intraabdominal	29 (19.6%)
Pelvic	7 (4.7%)
Chest wall	42 (28.4%)
Intrathoracic	9 (6.1%)
Head and neck	6 (4.1%)
Lower limb	8 (5.4%)
Upper limb	6 (4.1%)
DT focality, *n* (%)	​
Single	128 (86.5%)
Multifocal^c^	20 (13.5%)
Other DT locations, *n* (%)^a^	​
Abdominal wall	6 (30%)
Intraabdominal	8 (40%)
Pelvic	5 (25%)
Chest wall	3 (15%)
Lower limb	1 (5%)
Upper limb	1 (5%)
*CTNNB1* mutation status	​
Wild-type	3 (2%)
Mutated	7 (4.7%)
Not assessed/documented	138 (93.2%)
*APC* mutation status	​
Wild-type	37 (25%)
Mutated (known pathogenic variant)	18 (12.1%)
Variant of uncertain significance	1 (0.7%)
Not assessed/documented	92 (62.2%)
Family history of FAP	​
Yes	18 (12.2%)
Not documented	130 (87.8%)
Diagnosis of FAP	​
Yes	21 (14.2%)
Not documented	127 (85.8%)
History of other cancers, *n* (%)^a^	14 (9.5%)
Genitourinary cancers	4 (28.6%)
Sarcomas	3 (21.4%)
Breast cancer	2 (14.3%)
Colorectal cancer	2 (14.3%)
Melanoma and other skin cancers	1 (7.1%)
Others	2 (14.3%)

Abbreviation: SD, standard deviation.

a Patients may belong to more than one category.

b Primary tumor refers to the first tumor detected at diagnosis, or if >1 tumors were detected, the tumor that was largest in size.

c Multifocal refers to the presence of more than one noncontiguous tumor or lesion.

Of 56 patients (37.8%) who had documented *APC* gene testing results, 18 patients had known pathogenic variants of *APC*, representing 12.1% of the full patient cohort. Additionally, one patient reported a variant of uncertain significance. All 18 patients with *APC* pathogenic mutations and 3 patients who did not have any testing documentation of *APC* had a diagnosis of FAP (*n* = 21, 14.2%). A much smaller proportion of patients had documentation of *CTNNB1* testing (*n* = 10, 6.7%), including seven with *CTNNB1* mutation (4.7%) and three who were demonstrated to have the wild-type, nonmutated variant, which results in production of normal β-catenin (2%).

Among all patients, 88.5% (*n* = 131) had documentation of at least one episode of active disease, including 36.5% of patients (*n* = 54) with at least one episode of disease progression, 59.5% of patients (*n* = 88) with at least one episode of symptomatic disease, and 118 patients who received at least one line of treatment (79.7%; Supplementary Fig. S2). DT-related symptoms that were most documented in the clinical notes were tumor pain (*n* = 85, 57.4%), fullness (*n* = 23, 15.5%), loss of function (*n* = 22, 14.9%), and paresthesia (*n* = 17, 11.5%).

### Treatment patterns

The median number of lines of therapy received by patients was 1 (IQR: 1–3, range: 0–10), and 118 patients (79.7%) received at least one line of treatment. In the remaining 20.3% (*n* = 30) of patients with no documentation of DT treatment during the study period, 26 had regular DT-related hospital visits (with or without surveillance imaging) of at least 6-month intervals over a median follow-up period of 12.4 months.

**Figure 1.**
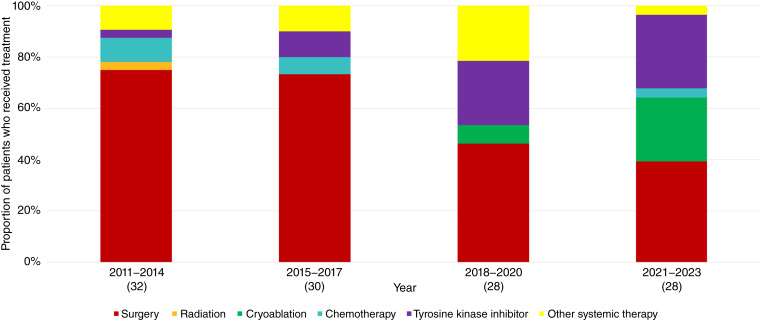
Trends of first-line treatment received by patients with DTs across time. Note: 30 patients received no active treatment, of which 26 had DT-related encounters of at least 6-month intervals during the study period. Overall first-line treatment patterns significantly changed over time (*P* = 0.003) based on Jonckheere–Terpstra tests for trend. Specifically, significant changes were observed for surgery (*P* < 0.001), cryoablation (*P* < 0.001), and TKIs (*P* < 0.001) but not for radiation (*P* = 0.15), chemotherapy (*P* = 0.18), and other systemic therapy (*P* = 0.94) based on Cochran–Armitage tests for trend.

Surgery was the most common treatment modality (*n* = 78, 52.7%), predominantly as the first line of treatment (*n* = 70/118, 59.3%; Table 2) but steadily decreasing over time (Fig. 1). During the study period, recurrence was noted among 27 of 78 (34.6%; 95% CI, 24.1%–45.2%) of patients after surgery with a median time to recurrence of 18.8 months (IQR: 11.5–34.1, range: 2.4–103.7). Other local treatment options observed in our study cohort included cryoablation (*n* = 33, 22.3%), radiotherapy (*n* = 7, 4.7%), transarterial embolization (*n* = 9, 6.1%), and ethanol ablation (*n* = 1, 0.7%), primarily in later lines of therapy.

**Table 2.** Treatment patterns of patients with DT during the entire disease course and up to the fourth line of therapy.

Treatment	Overall	Line of therapy			
		1	2	3	4
*N*	148	118	55	40	18
No active treatment, *n* (%)^a^	30 (20.3%)	—	—	—	—
With only active surveillance^a^	26 (17.6%)	​	​	​	​
Surgery, *n* (%)	78 (52.7%)	70 (59.3%)	8 (14.5%)	3 (7.5%)	2 (11.1%)
Radiation, *n* (%)	7 (4.7%)	1 (0.8%)	2 (3.6%)	4 (10%)	0 (0%)
Cryoablation, *n* (%)	33 (22.3%)	9 (7.6%)	8 (14.5%)	14 (35%)	4 (22.2%)
Chemotherapy, *n* (%)	19 (12.8%)	6 (5.1%)	10 (18.2%)	3 (7.5%)	2 (11.1%)
Liposomal doxorubicin	11 (7.4%)	3 (2.5%)	5 (9.1%)	1 (2.5%)	1 (5.6%)
Methotrexate + vinblastine	12 (8.1%)	3 (2.5%)	5 (9.1%)	2 (5%)	1 (5.6%)
Doxorubicin + dacarbazine	1 (0.7%)	0 (0%)	0 (0%)	0 (0%)	0 (0%)
TKI, *n* (%)	41 (27.7%)	19 (16.1%)	15 (27.3%)	11 (27.5%)	5 (27.8%)
Imatinib	15 (10.1%)	2 (1.7%)	6 (10.9%)	4 (10%)	2 (11.1%)
Pazopanib	6 (4.1%)	0 (0%)	3 (5.5%)	1 (2.5%)	0 (0%)
Sorafenib	33 (22.3%)	17 (14.4%)	6 (10.9%)	6 (15%)	3 (16.7%)
Other systemic therapy, *n* (%)	27 (18.2%)	13 (11%)	7 (12.7%)	4 (10%)	5 (27.8%)
Tamoxifen	4 (2.7%)	2 (1.7%)	1 (1.8%)	0 (0%)	0 (0%)
Tamoxifen + NSAIDs	24 (16.2%)	11 (9.3%)	5 (9.1%)	4 (10%)	4 (22.2%)
Clinical trial	1 (0.7%)	0 (0%)	1 (1.8%)	0 (0%)	1 (5.6%)
Hydroxyurea	2 (1.4%)	0 (0%)	0 (0%)	0 (0%)	0 (0%)
Others, *n* (%)	10 (6.8%)	0 (0%)	5 (9.1%)	1 (2.5%)	0 (0%)
DEB-TACE	6 (4.1%)	0 (0%)	4 (7.3%)	1 (2.5%)	0 (0%)
TACE	3 (2%)	0 (0%)	1 (1.8%)	0 (0%)	0 (0%)
Ethanol ablation	1 (0.7%)	0 (0%)	0 (0%)	0 (0%)	0 (0%)

Abbreviations: DEB, drug-eluting bead; TACE, transarterial chemoembolization.

a No active treatment refers to patients with no documented treatment since DT diagnosis until the end of the study period. A subgroup of patients received regular monitoring for DT (based on DT-related hospital encounters) at intervals of not more than 6 months and are described as having received active surveillance.

Of the systemic treatment options available during the study period, TKIs were the most common type used at any line of treatment (*n* = 41, 27.7%), including sorafenib (*n* = 33, 22.3%), imatinib (*n* = 15, 10.1%), and pazopanib (*n* = 6, 4.1%), whereas chemotherapy was utilized by 19 patients (12.8%; Table 2). Patients may have received different TKIs at different lines of treatment. Patients who received systemic therapy at first-line treatment tended to have tumors that were larger in size at DT diagnosis compared with those who received locoregional therapy (*P* = 0.005; Supplementary Table S3).

The proportion of patients started on different first-line treatment modalities each year changed significantly across the study period (*P* = 0.003). Specifically, there was a significant decrease in the use of surgery in the first-line setting (Fig. 1) from 75% (24/32) during 2011 to 2014 and 73.3% (22/30) during 2015 to 2017 and 46.4% (13/28) during 2018 to 2020 and 39.3% (11/28) during 2021 to 2023 (*P* < 0.001). This was accompanied by an increase in the use of TKIs from 3.1% (1/32) during 2011 to 2014 and 10% (3/30) during 2015 to 2017 to 25% (7/28) during 2018 to 2020 and 28.6% (8/28) during 2021 to 2023 (Fig. 1; *P* < 0.001). Use of cryoablation as first-line treatment started in 2018 and significantly increased from 7.1% (2/28) during 2018 to 2020 and 28.6% (8/28) during 2021 to 2023 (Fig. 1; *P* < 0.001).

### Healthcare resource utilization

A summary of healthcare resource utilization stratified by disease activity is shown in Table 3. 88.5% of patients had at least one outpatient visit related to DTs, with an average number of 2.4 visits per patient per year. In contrast, only a small proportion of patients had emergency visits related to DTs (*n* = 5, 3.4%). Approximately one fifths of patients (*n* = 30, 20.3%) required hospitalizations related to DTs with a median length of stay per year of 4.5 days (IQR: 3–8, range: 1–35).

**Table 3.** Healthcare resource utilization related to DTs stratified by the time since diagnosis and disease state.

Healthcare resource utilization related to DT	Time period		
	Overall (*n* = 148)	During symptomatic disease (*n* = 88)	During active disease (*n* = 131)
% of patients with outpatient visits, *n* (%)^a^	131 (88.5%)	74 (84.1%)	84 (64.1%)
Outpatient visits PPPY, median (IQR, range)^a^	2.4 (0.5–6.3, 0–24.2)	7.2 (3.1–18.6, 0–54.1)	4.7 (0–18.2, 0–69.6)
% of patients with emergency room visits, *n* (%)	5 (3.4%)	4 (4.5%)	3 (2.3%)
Emergency room visits PPPY, median (IQR, range)	0 (0–0, 0–0.97)	0 (0–0, 0–1.18)	0 (0–0, 0–0.70)
% of patients with hospitalizations, *n* (%)	30 (20.3%)	9 (10.2%)	18 (13.7%)
Length of hospitalization in days, median (IQR, range)^b^	4.5 (3–8, 1–35)	6 (6–7, 2–13)	4.5 (2–6, 1–7)

Abbreviations: PPPY, per patient per year; SD, standard deviation.

a Refers to office visits in outpatient setting.

b Among patients with hospitalizations.

An increase in healthcare resource utilization related to DTs was observed during periods of symptomatic disease and active disease, compared with the overall study period. Although there was no increase in the proportion of patients who engaged outpatient services, the median number of outpatient visits almost tripled in the event of symptomatic disease (7.2 per patient per year) and doubled during active disease (4.7 per patient per year).

### Potential misdiagnosis prior to DT diagnosis

A total of 43 patients (29.1%) potentially had a misdiagnosis prior to a final confirmed diagnosis of DT based on tissue histology (Table 4). Most common misdiagnoses included other benign tumors (*n* = 31, 20.3%), low-grade sarcomas (*n* = 11, 7.2%), nodular fasciitis (*n* = 4, 2.6%), and procedure-related scars (*n* = 3, 2%). Compared with patients with DTs and no misdiagnosis, those who had a prior misdiagnosis had a similar median age at DT diagnosis (39 vs. 35 years). Patients with prior misdiagnoses tended to have a more complex medical history than those with no misdiagnosis, with a higher proportion of patients carrying *APC* mutations (32.6% vs. 4.8%, *P* < 0.001), having a diagnosis of FAP (39.5% vs. 3.8%, *P* < 0.001) and a prior history of other malignancies (20.9% vs. 4.5%, *P* = 0.002). At diagnosis of DTs, patients were more likely to have multifocal disease (25.6% vs. 8.6%, *P* = 0.006) and intraabdominal or intrathoracic tumors (intraabdominal: 34.9% vs. 13.3%, intrathoracic: 18.6% vs. 1%, *P* < 0.001). In the first year prior to DT diagnosis, healthcare resource utilization was higher among patients with prior misdiagnoses, with a higher proportion of patients requiring outpatient visits (65.1% vs. 30.3%, *P* < 0.001), emergency room visits (14% vs. 2.6%, *P* = 0.03), and hospitalization (25.6% vs. 2.6%, *P* < 0.001; Table 4).

**Table 4.** Patient characteristics and healthcare resource utilization associated with DT misdiagnosis.

Parameter	*N* = 148
Proportion of patients with potential DT misdiagnosis, *n* (%)	43 (28.1%)
Potential misdiagnosis, *n* (%)	​
Procedure-related scars	3 (2%)
Nodular fasciitis	4 (2.6%)
Other benign tumors	31 (20.3%)
Low-grade sarcomas	11 (7.2%)
Earliest misdiagnosis to DT diagnosis in years, median (IQR, range)	0.8 (0.1–1.7, 0.1–2)

Patient characteristics	Potential misdiagnosis (*n* = 43)	No potential misdiagnosis (*n* = 105)	*P* value^a^
Age at diagnosis, mean (SD)	39.5 (13.8)	37.5 (14.3)	0.44
Age at diagnosis, median (IQR, range)	39 (29–48, 18–78)	35 (27–46, 18–76)	0.27
Sex, *n* (%)	​	​	0.09
Male	18 (41.9%)	29 (27.6%)	​
Female	25 (58.1%)	76 (72.4%)	​
Undocumented	​	​	​
Race and ethnicity, *n* (%)^b^	​	​	0.05
Asian	3 (7%)	0 (0%)	​
Black/African American	1 (2.3%)	2 (1.9%)	​
Hispanic/Latino	4 (9.3%)	7 (6.7%)	​
Native American/Alaska Native	1 (2.3%)	0 (0%)	​
White	35 (81.4%)	96 (91.4%)	​
Undocumented	1 (2.3%)	4 (3.8%)	​
Insurance status, *n* (%)	​	​	0.47
Commercial	33 (76.7%)	85 (81%)	​
Medicare	4 (9.3%)	6 (5.7%)	​
Medicaid	4 (9.3%)	6 (5.7%)	​
VA/Tricare	2 (4.7%)	3 (2.9%)	​
Uninsured	0 (0%)	5 (4.8%)	​
Geographic region, *n* (%)	​	​	**0.01**
Utah	33 (76.7%)	57 (54.3%)	​
Non-Utah	10 (23.3%)	48 (45.7%)	​
Primary DT size in mm, mean (SD)^c^	76.8 (42.4)	64.2 (34)	0.07
Primary DT size in mm, median (IQR, range)	70 (42–103, 18–180) (Unreported = 4)	56 (38.5–88.5, 11–170) (Unreported = 5)	0.15
Primary DT location, *n* (%)^c^	​	​	**<0.001**
Abdominal wall	11 (25.6%)	30 (28.6%)	​
Intraabdominal	15 (34.9%)	14 (13.3%)	​
Pelvic	1 (2.3%)	6 (5.7%)	​
Chest wall	6 (14%)	36 (34.3%)	​
Intrathoracic	8 (18.6%)	1 (1%)	​
Head and neck	0 (0%)	6 (5.7%)	​
Lower limb	1 (2.3%)	7 (6.7%)	​
Upper limb	1 (2.3%)	5 (4.8%)	​
DT focality, *n* (%)	​	​	**0.006**
Single	32 (74.4%)	96 (91.4%)	​
Multifocal^d^	11 (25.6%)	9 (8.6%)	​
*CTNNB1* mutation status	​	​	0.11
Wild-type	0 (0%)	3 (2.9%)	​
Mutated	0 (0%)	8 (6.7%)	​
Not assessed/documented	43 (100%)	99 (90%)	​
*APC* mutation status	​	​	**<0.001**
Wild-type	7 (16.3%)	30 (28.6%)	​
Mutated	14 (32.6%)	5 (4.8%)	​
Not assessed/documented	22 (51.2%)	72 (66.7%)	​
Family history of FAP	​	​	**<0.001**
Yes	14 (32.6%)	4 (3.8%)	​
Not documented	29 (67.4%)	101 (96.2%)	​
Diagnosis of FAP	​	​	**<0.001**
Yes	17 (39.5%)	4 (3.8%)	​
Not documented	26 (60.5%)	101 (96.2%)	​
History of other cancers, *n* (%)	9 (20.9%)	5 (4.5%)	**0.002**
Overall healthcare resource utilization	​	​	​
Within the first year before DT diagnosis	(*n* = 43)	(*n* = 76)	​
% of patients with outpatient visits, *n* (%)^e^	28 (65.1%)	23 (30.3%)	**<0.001**
Outpatient visits PPPY, median (IQR, range)^e^	3 (0–14, 0–118)	0 (0–4.3, 0–73)	**0.002**
% of patients with emergency room visits, *n* (%)	6 (14%)	2 (2.6%)	**0.03**
Emergency room visits PPPY, median (IQR, range)	0 (0–0, 0–4)	0 (0–0, 0–12)	**0.02**
% of patients with hospitalizations, *n* (%)	11 (25.6%)	2 (2.6%)	**<0.001**
Length of hospitalization in days, median (IQR, range)^f^	4 (3–13, 2–17)	13.5 (11–16, 11–16)	0.11
Between the first and second years before DT diagnosis	(*n* = 26)	(*n* = 19)	​
% of patients with outpatient visits, *n* (%)^e^	12 (46.2%)	8 (10.5%)	0.51
Outpatient visits PPPY, median (IQR, range)^e^	0 (0–17, 0–80)	0 (0–5.3, 0–60)	0.48
% of patients with emergency room visits, *n* (%)	1 (3.8%)	3 (3.9%)	0.30
Emergency room visits PPPY, median (IQR, range)	0 (0–0, 0–1)	0 (0–0, 0–2)	0.35
% of patients with hospitalizations, *n* (%)	6 (23.1%)	3 (3.9%)	0.71
Length of hospitalization in days, median (IQR, range)^f^	10.5 (2–20, 2–40)	5 (3–13, 3–13)	0.60

Bold *P* values indicate statistical signficance, i.e., *P* < 0.05.

a *P* values are from comparison of characteristics between patients with potential misdiagnosis and those without using Wilcoxon rank-sum tests for continuous variables and *χ*^2^ tests/Fisher exact test for categorical variables.

b Patients may belong to more than one category.

c Primary tumor refers to the first tumor detected at diagnosis, or if >1 tumors were detected, the tumor that was largest in size.

d Multifocal refers to the presence of more than one noncontiguous tumor or lesion.

e Refers to office visits in outpatient setting.

f Among patients with hospitalization.

## Discussion

Our study adds to the limited literature on DTs by characterizing the patient journey in DT from diagnosis to treatment based on electronic health records from an academic center in the United States. Notably, this study is one of a few that have been published to date to describe healthcare resource utilization among patients with DTs (20). Additionally, the use of a chart review approach allowed us to collect comprehensive data of the patient journey, including types of treatment used in the community prior to referral to our center based on referral documentation. In summary, DTs were associated with substantial symptom burden in a notable proportion of patients, and approximately one thirds of patients with DTs may have been misdiagnosed before a confirmed diagnosis of DTs. Episodes of symptomatic disease and misdiagnosis were associated with a higher rate of healthcare resource utilization, and treatment patterns for DTs have changed in the last decade.

Approximately half of the patients in our study who had treatment received surgery as the first active intervention, similar to findings of some other observational studies in the United States (48%), Italy (50%), United Kingdom (51%), and India (58%; refs. 21–24). However, due to the low incidence of DTs and to accrue a sizable patient cohort, the data period of most studies extended beyond the past decade, and treatment guidelines have since evolved. Some studies using more recent data in Japan, Europe, and the United States reported a steady decline in surgery rates similar to ours (24, 25). For example, a multinational study in Europe (2012–2018) reported that only 16% of patients who required treatment received surgery (26). These observations reflect the shift away from a predominantly surgical approach to systemic treatment for active intervention to manage DTs as recommended by treatment guidelines since 2015 (8, 11, 12). In our study, surgical rates have steadily decreased during the study period and were 39.3% during 2021 to 2023.

As surgery rates declined, the utilization of systemic treatments increased; however, the preferred systemic treatment approaches differed across studies. An increase in the use of TKIs was observed in our study after phase III trial data supporting the use of sorafenib in progressive or symptomatic DTs were reported (19). Cytotoxic chemotherapy, nonsteroidal anti-inflammatory drugs (NSAID) alone and hormonal treatment were the predominant systemic treatment approach noted in other recently published studies based in the United States and Europe (21, 22, 26). Notably, the observation period of these studies started before 2015, and NSAIDs and hormonal treatment are no longer recommended for DT treatment. Taken together with the finding that patients with DTs often require multiple lines of therapy, there is a need for more targeted DT treatment options. Improved understanding of treatment sequencing and outcomes is also necessary to optimize treatment options based on clinical data and patient-specific characteristics, including tumor size, location, and associated mutations.

Due to the nonspecific nature of DT-related symptoms, similarities in histology to other myofibroblastic disorders, historic absence of a specific ICD-CM code, and the rarity of DTs, misdiagnosis is a potential cause of delay in the journey to DT diagnosis (27, 28). We found that a notable proportion of patients may have previously been misdiagnosed with other conditions, including low-grade sarcomas and other benign soft tissue tumors. Compared with those without misdiagnoses, more patients with prior misdiagnoses had a diagnosis of FAP, which explains the increased likelihood of multifocal disease. Whereas the index of suspicion is high for DTs among patients with FAP, misdiagnosis is still possible as FAP also increases the risk of other benign tumors histologically and clinically similar to DTs. Patients with misdiagnosis were also more likely to have larger tumors when diagnosed with DTs. A potential explanation is that the delay in DT diagnosis may have prevented timely initiation of DT-specific care and resulted in tumor growth. Additionally, our findings have demonstrated increased healthcare resource use in the year prior to DT diagnosis among patients with prior misdiagnoses compared with those without, reflecting the additional diagnostic resources used.

Past studies have shown that misdiagnosis of DTs, particularly if patients were misdiagnosed with a more severe condition such as cancer, can lead to substantial distress (5). The negative impact on patients, the delayed initiation of appropriate disease management, and the unnecessary use of healthcare resources underscore the importance of a timely and accurate diagnosis and shortening the diagnostic journey for patients with DTs, which can be achieved by improving the awareness of DTs among healthcare providers. Maintaining a strong and consistent patient–provider relationship is also crucial to allow continuous, uninterrupted follow-up and monitoring, which will not only shorten any delays in diagnosis but also ensure compliance and adequate assessment of treatment benefits and adverse effects. Diagnostic methods, such as *CTNNB1* testing, may also shorten the diagnostic journey. *CTNNB1* mutations are found in more than 80% of sporadic DTs (27). Therefore, *CTNNB1* testing is useful to diagnose or rule out DTs when *APC* mutations are not detected or morphologic features of DTs are inconclusive in the biopsy specimen (29). Notably, the proportion of patients with *CTNNB1* testing was low in our participant cohort, potentially due to cost and reimbursement issues. There is also currently a lack of evidence to suggest that *CTNNB1* mutation status is predictive of treatment response to systemic therapies or survival outcomes (21, 30). Recently, the addition of ICD-10-CM codes specific to DTs could also support a timely and accurate diagnosis.

DTs are associated with substantial symptom burden in a large proportion of patients, which may be an indicator of poor disease control. Tumor-related pain was the most common symptom reported in our study, and the prevalence of pain during the course of the disease was similar to that reported in Rigaux and colleagues (31), which specifically examined pain burden among patients with DTs. Penel and colleagues (32) reported a lower prevalence of pain; however, pain in this study was measured only at diagnosis; hence, pain that developed later in the disease course would not have been captured. Although not always indicative of radiographic progression or increasing DT size, high symptom burden can lead to deteriorating quality of life, whereas more intense pain at diagnosis has been found to be associated with worse event-free survival (2, 32). Healthcare resource utilization among patients with DTs has not been comprehensively reported to date, but our findings show more frequent outpatient office visits during periods of symptomatic disease compared with the overall follow-up period. Therefore, better strategies for symptom management in DTs, including more effective treatment with less toxicity, may be needed both to improve the patient experience and for more efficient use of healthcare resources.

The findings of our study should be interpreted in the context of several limitations. First, data were collected from a single study site in the United States and may not be representative of the entire disease journey of the patient or overall DT population. However, the HCI is a referral center for sarcoma and is expected to serve most patients with DTs in Utah and other Mountain West states within its outreach area. Given that patients are referred to the HCI for DTs that are more complex to manage or after progression on prior active surveillance in community centers, the treatment patterns observed in our study could potentially be different from those in other centers. Due to the low incidence of DTs, a single study site also limited our sample size and the ability to make meaningful comparisons between patient subgroups. Additionally, due to the retrospective nature of our study, data collected on symptomatic disease were highly dependent on whether patients volunteer information during routine visits and whether medical history was well-documented as part of routine care. Therefore, reports of symptomatic disease were likely to be underestimated; nevertheless, the findings in our study indicate that at least half of patients with DTs reported tumor-related pain. The retrospective nature of our study also prevented the capture of active surveillance, as the lag period prior to treatment may be due to various reasons, which are not always comprehensively documented in the clinical notes. Additionally, healthcare resource utilization was presented descriptively and not adjusted for comorbidity burden as the data were not collected. However, we only included episodes of care that were tagged with ICD billing codes 238.1 and D48.1, which were conventionally used for DT prior to October 2023. The use of these billing codes suggests that management of DTs may have been part of these episodes of care, whether the primary reason of admission was DT-related.

The occurrence of potential misdiagnosis of DTs and the associated increased healthcare resource utilization highlights the need for timely and accurate diagnosis, which may be achieved with improved awareness of DTs among healthcare providers. Treatment patterns for DTs have changed in the last decade in line with recommendations to limit the use of surgery. However, the varied treatment approaches used in place of surgery, multiple lines of therapy, and substantial symptom burden among some patients support the use of better therapeutic agents. FDA approval of nirogacestat may transform the treatment landscape of DTs, and future work is needed to examine how DT management and patient outcomes may change.

## Supplementary Material

Supplementary Figure S1 Patient flow diagram showing how the study cohort was identified from the data source

Supplementary Figure S2 Venn diagram depicting the overlap between patients with different criteria defining active disease

Supplementary Table S1 List of terms used in the study to infer disease progression among study participants from documentation in clinical notes

Supplementary Table S2 List of ICD-CM codes used to identify patients with potential misdiagnoses

Supplementary Table S3 Additional data showing the baseline characteristics of patients stratified by the type of first line treatment received

## Data Availability

The data generated in this study are not publicly available due to privacy or ethical restrictions but are available upon reasonable request to the corresponding author.
