# Supplementary material for: Real-World Characterization and Treatment Patterns of Patients with Desmoid Tumors at an Academic Center in the United States
Source: Cancer Res Commun. 2026 Apr 9;6(4):792–802. doi: 10.1158/2767-9764.CRC-25-0581 (PMC13063223; doi:10.1158/2767-9764.CRC-25-0581)
Supplement: Supplementary Figure S1 — Patient flow diagram showing how the study cohort was identified from the data source [file crc-25-0581_supplementary_figure_s1_suppsf1.pdf]

Supplementary Figure S1 Patient flow diagram

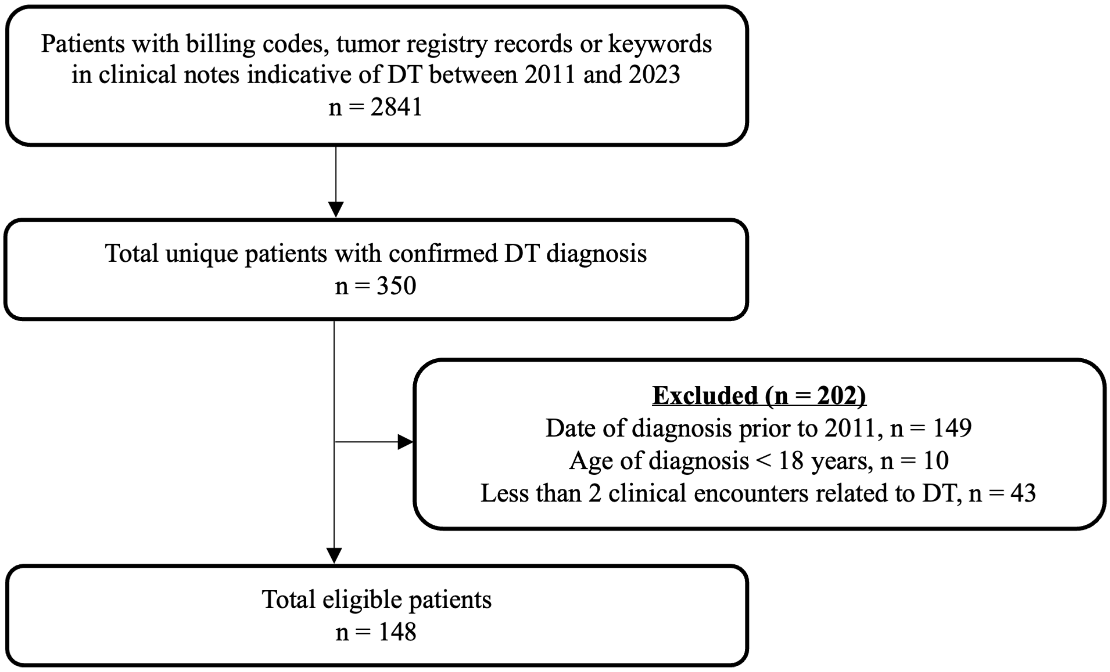

Abbreviations: DT, desmoid tumor; ED, emergency department; HCI-TR, Huntsman Cancer Institute-Tumor Registry; ICD, International Classification of Disease; LTFU, lost to follow-up
