# Supplementary material for: Real-World Characterization and Treatment Patterns of Patients with Desmoid Tumors at an Academic Center in the United States
Source: Cancer Res Commun. 2026 Apr 9;6(4):792–802. doi: 10.1158/2767-9764.CRC-25-0581 (PMC13063223; doi:10.1158/2767-9764.CRC-25-0581)

Supplementary Figure S2 Patients with active disease, stratified by study criteria (Active treatment, disease progression and/or symptomatic disease)

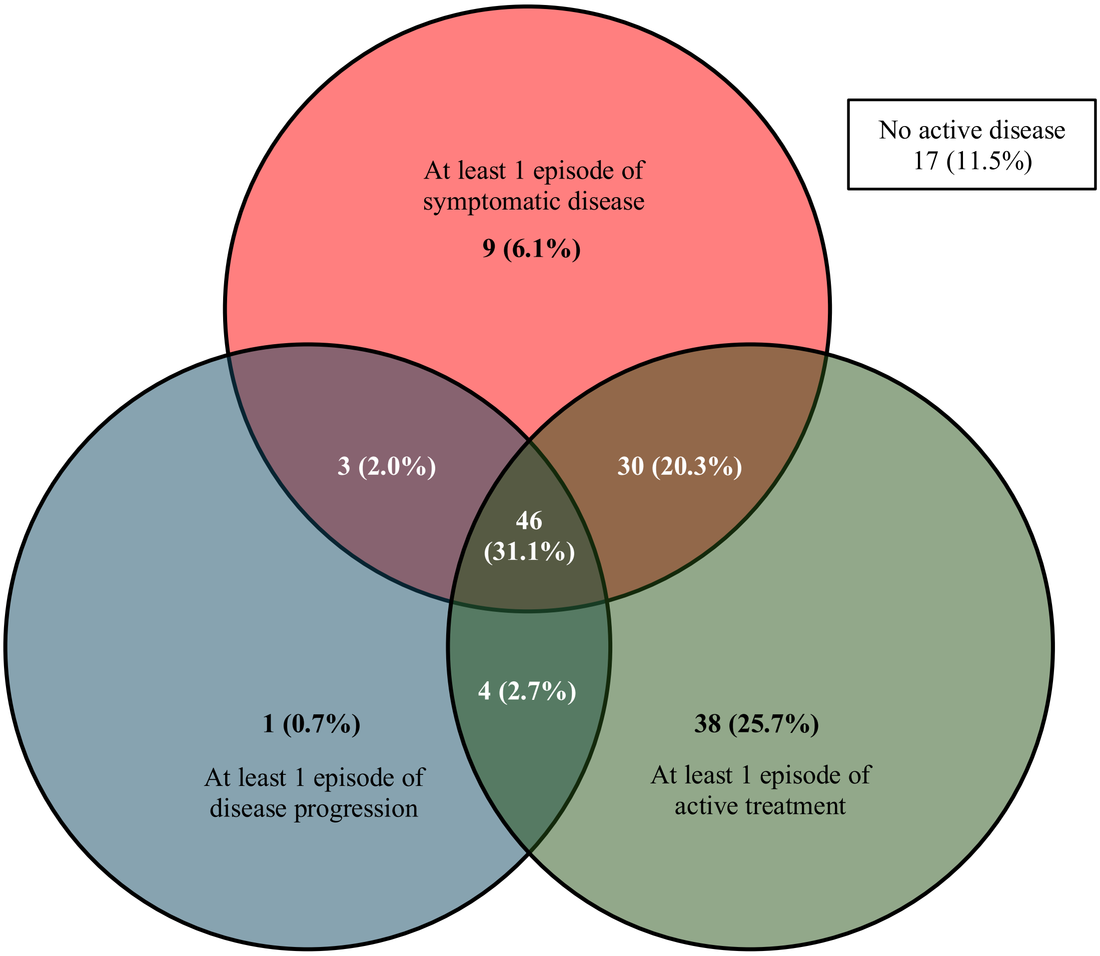

Supplement: Supplementary Figure S2 — Venn diagram depicting the overlap between patients with different criteria defining active disease [file crc-25-0581_supplementary_figure_s2_suppsf2.pdf]
