# Supplementary material for: Real-World Characterization and Treatment Patterns of Patients with Desmoid Tumors at an Academic Center in the United States
Source: Cancer Res Commun. 2026 Apr 9;6(4):792–802. doi: 10.1158/2767-9764.CRC-25-0581 (PMC13063223; doi:10.1158/2767-9764.CRC-25-0581)
Supplement: Supplementary Table S1 — List of terms used in the study to infer disease progression among study participants from documentation in clinical notes [file crc-25-0581_supplementary_table_s1_suppst1.pdf]

Supplementary Table S1 Terms in clinical notes for inference of disease progression

	Example of terms
Disease progression	“progression”, “worsening”, “worsened”, “increase in tumor size”, “tumor growth”, “new lesion/tumor”
