# Supplementary material for: Real-World Characterization and Treatment Patterns of Patients with Desmoid Tumors at an Academic Center in the United States
Source: Cancer Res Commun. 2026 Apr 9;6(4):792–802. doi: 10.1158/2767-9764.CRC-25-0581 (PMC13063223; doi:10.1158/2767-9764.CRC-25-0581)
Supplement: Supplementary Table S2 — List of ICD-CM codes used to identify patients with potential misdiagnoses [file crc-25-0581_supplementary_table_s2_suppst2.pdf]

Supplementary Table S2 ICD-CM codes for identification of potential misdiagnoses

Potential misdiagnosis	ICD-CM code	Code definition
Hypertrophic and keloid scars	701.4 (ICD-9)	Hypertrophic scars
	L91.0 (ICD-10)	Keloid scars
Procedure-related scars	709.2 (ICD-9)	Scar conditions and fibrosis of skin
	L90.5 (ICD-10)	Scar conditions and fibrosis of skin
Nodular fasciitis	728.79 (ICD-9)	Fibromatoses of muscle, ligament, and fascia
	M72.4 (ICD-10)	Pseudosarcomatous fibromatosis
Fibromas	210 - 229 (ICD-9)	Benign neoplasms
Lipomas	D10 - D36 (ICD-10)	Benign neoplasms
Smooth muscle tumors (Leiomyoma)		
Nerve sheath tumors (Schwannoma)		
Low-grade sarcomas	171 (ICD-9)	Malignant neoplasm of other connective and soft tissue
Gastrointestinal stromal tumor		
	C49 (ICD-10)	Malignant neoplasm of other connective and soft tissue
