# Supplementary material for: Real-World Characterization and Treatment Patterns of Patients with Desmoid Tumors at an Academic Center in the United States
Source: Cancer Res Commun. 2026 Apr 9;6(4):792–802. doi: 10.1158/2767-9764.CRC-25-0581 (PMC13063223; doi:10.1158/2767-9764.CRC-25-0581)
Supplement: Supplementary Table S3 — Additional data showing the baseline characteristics of patients stratified by the type of first line treatment received [file crc-25-0581_supplementary_table_s3_suppst3.pdf]

Supplementary Table S3 Baseline characteristics of patients stratified by first line therapy

Characteristic	Surgery (n = 70)	Radiation (n = 1)	Cryoablation (n = 9)	Chemotherapy (n = 6)	TKIs (n = 19)	Other systemic therapy (n=13)	p-value§
Age at diagnosis, median (IQR)	36.3 (28.6-46.5)	34.6 (-)	35.0 (25.6-59.2)	27.2 (22.6-37.1)	33.4 (25.6-62.6)	29.7 (27.0-39.3)	0.24
Sex, n (%)							0.46
Male	21 (30.0%)	0 (0.0%)	3 (33.3%)	3 (50.0%)	6 (31.6%)	5 (38.5%)	
Female	49 (70.0%)	1 (100.0%)	6 (66.7%)	3 (50.0%)	13 (68.4%)	8 (61.5%)	
Race and ethnicity, n (%)†							0.37
Asian	3 (4.3%)	0 (0.0%)	0 (0.0%)	0 (0.0%)	0 (0.0%)	0 (0.0%)	
Black/African-American	2 (2.9%)	0 (0.0%)	0 (0.0%)	0 (0.0%)	0 (0.0%)	1 (7.7%)	
Hispanic/Latino	4 (5.7%)	0 (0.0%)	1 (11.1%)	0 (0.0%)	2 (10.5%)	2 (15.4%)	
Native American/Alaska Native	0 (0.0%)	0 (0.0%)	0 (0.0%)	0 (0.0%)	1 (5.3%)	0 (0.0%)	
White	64 (91.4%)	1 (100.0%)	8 (88.9%)	6 (100.0%)	16 (84.2%)	11 (84.6%)	
Undocumented	0 (0.0%)	0 (0.0%)	1 (11.1%)	0 (0.0%)	1 (5.3%)	0 (0.0%)	
Insurance status, n (%)							0.85
Commercial	53 (75.7%)	1 (100.0%)	8 (88.9%)	6 (100.0%)	14 (73.7%)	9 (69.2%)	
Medicare	6 (8.6%)	0 (0.0%)	1 (11.1%)	0 (0.0%)	2 (10.5%)	0 (0.0%)	
Medicaid	6 (8.6%)	0 (0.0%)	0 (0.0%)	0 (0.0%)	1 (5.3%)	3 (23.1%)	
VA/Tricare	2 (2.9%)	0 (0.0%)	0 (0.0%)	0 (0.0%)	2 (10.5%)	0 (0.0%)	
Uninsured	3 (4.3%)	0 (0.0%)	0 (0.0%)	0 (0.0%)	0 (0.0%)	1 (7.7%)	
Geographic region, n (%)							0.54
Utah	45 (64.3%)	0 (0.0%)	6 (66.7%)	4 (66.7%)	11 (57.9%)	7 (53.8%)	
Non-Utah	25 (35.7%)	1 (100.0%)	3 (33.3%)	2 (33.3%)	8 (42.1%)	6 (46.2%)	
Primary DT size in mm, median (IQR, range)	56.5 (36.0-91.0) (UR = 6)	35.0 (-)	45.0 (26.0-77.0)	80.5 (62.0-108.0)	87.0 (55.0-107.0)	66.0 (50.0-110.0) (UR = 1)	**0.005**

Characteristic	Surgery (n = 70)	Radiation (n = 1)	Cryoablation (n = 9)	Chemotherapy (n = 6)	TKIs (n = 19)	Other systemic therapy (n=13)	p-value§
Primary DT location, n (%)‡							0.47
Abdominal wall	16 (22.9%)	0 (0.0%)	4 (44.4%)	1 (16.7%)	4 (21.1%)	2 (15.4%)	
Intra-abdominal	15 (21.4%)	0 (0.0%)	1 (11.1%)	1 (16.7%)	2 (10.5%)	3 (23.1%)	
Pelvic	4 (5.7%)	0 (0.0%)	0 (0.0%)	1 (16.7%)	2 (10.5%)	0 (0.0%)	
Chest wall	23 (32.9%)	0 (0.0%)	2 (22.2%)	1 (16.7%)	6 (31.6%)	3 (23.1%)	
Intra-thoracic	4 (5.7%)	0 (0.0%)	0 (0.0%)	1 (16.7%)	1 (5.3%)	2 (15.4%)	
Head and neck	2 (2.9%)	0 (0.0%)	1 (11.1%)	1 (16.7%)	1 (5.3%)	0 (0.0%)	
Lower limb	2 (2.9%)	0 (0.0%)	1 (11.1%)	0 (0.0%)	3 (15.8%)	2 (15.4%)	
Upper limb	4 (5.7%)	1 (100.0%)	0 (0.0%)	0 (0.0%)	0 (0.0%)	1 (7.7%)	
DT focality, n (%)							0.08
Single	62 (88.6%)	1 (100.0%)	8 (88.9%)	6 (100.0%)	14 (73.7%)	9 (69.2%)	
Multifocal^	8 (11.4%)	0 (0.0%)	1 (11.1%)	0 (0.0%)	5 (26.3%)	4 (30.8%)	
CTNNB1 mutation status							0.86
Wild-type	0 (0.0%)	0 (0.0%)	1 (11.1%)	0 (0.0%)	1 (5.3%)	0 (0.0%)	
Mutated	4 (5.7%)	0 (0.0%)	0 (0.0%)	0 (0.0%)	2 (10.5%)	0 (0.0%)	
Not assessed/documented	66 (94.3%)	1 (100.0%)	8 (88.9%)	6 (100.0%)	16 (84.2%)	13 (100.0%)	
APC mutation status							0.22
Wild-type	19 (27.1%)	0 (0.0%)	1 (11.1%)	3 (50.0%)	8 (42.1%)	2 (15.4%)	
Mutated	5 (7.1%)	0 (0.0%)	1 (11.1%)	0 (0.0%)	2 (10.5%)	4 (30.8%)	
Variant of uncertain significance	1 (1.4)	0 (0.0%)	0 (0.0%)	0 (0.0%)	0 (0.0%)	0 (0.0%)	
Not assessed/documented	45 (64.3%)	1 (100.0%)	7 (77.8%)	3 (50.0%)	9 (47.4%)	7 (53.8%)	
Family history of FAP							0.16
Yes	65 (92.9%)	1 (100.0%)	8 (88.9%)	6 (100.0%)	18 (94.7%)	8 (61.5%)	
Not documented	5 (7.1%)	0 (0.0%)	1 (11.1%)	0 (0.0%)	1 (5.3%)	5 (38.5%)	
Clinical diagnosis of FAP							0.20
Yes	63 (90.0%)	1 (100.0%)	8 (88.9%)	6 (100.0%)	17 (89.5%)	8 (61.5%)	
Not documented	7 (10.0%)	0 (0.0%)	1 (11.1%)	0 (0.0%)	2 (10.5%)	5 (38.5%)	

Characteristic	Surgery (n = 70)	Radiation (n = 1)	Cryoablation (n = 9)	Chemotherapy (n = 6)	TKIs (n = 19)	Other systemic therapy (n=13)	p-value§
History of other cancers, n (%)†	7 (10.0%)	0 (0.0%)	1 (11.1%)	0 (0.0%)	1 (5.3%)	3 (23.1%)	0.93
Genitourinary cancers	2 (28.6%)	0 (0.0%)	0 (0.0%)	0 (0.0%)	1 (100%)	0 (0.0%)	
Sarcomas	2 (28.6%)	0 (0.0%)	0 (0.0%)	0 (0.0%)	0 (0.0%)	1 (33.3%)	
Breast cancer	2 (28.6%)	0 (0.0%)	0 (0.0%)	0 (0.0%)	0 (0.0%)	0 (0.0%)	
Colorectal cancer	0 (0.0%)	0 (0.0%)	1 (100%)	0 (0.0%)	0 (0.0%)	1 (33.3%)	
Melanoma or other skin cancers	1 (14.3%)	0 (0.0%)	0 (0.0%)	0 (0.0%)	0 (0.0%)	0 (0.0%)	
Others	0 (0.0%)	0 (0.0%)	0 (0.0%)	0 (0.0%)	0 (0.0%)	1 (33.3%)	

† Patients may belong to more than 1 category

‡ Primary tumor refers to the first tumor detected at diagnosis or if >1 tumors were detected, the tumor that was largest in size.

^ Multifocal refers to the presence of more than 1 non-contiguous tumor or lesion.

§ p-values are from comparison of characteristics between patients who received locoregional treatment modalities (surgery, radiation and cryoablation) and those who received systemic treatment modalities (chemotherapy, TKIs, other systemic therapy) in the first line setting using Wilcoxon rank sum tests for continuous variables and Chi-squared tests/Fisher's exact test for categorical variables.

Abbreviations: *APC* - adenomatous polyposis coli gene; *CTNNB1*- catenin beta-1 gene; DT – desmoid tumor; FAP – familial adenomatous polyposis; SD - standard deviation; UR - Unreported.
